# Quantitative Analysis of the Effects of Irrigation Frequency Under Constant Total Irrigation Amount on Photosynthetic Accumulation, Source–Sink Coordination, and Water–Grain–Quality Synergy in Wide-Row Precision-Sown Winter Wheat

**DOI:** 10.3390/plants15142115

**Published:** 2026-07-08

**Authors:** Shengfeng Wang, Enlai Zhan, Guowei Liang, Zijun Long, Xiaobei Feng

**Affiliations:** School of Water Conservancy, North China University of Water Resources and Electric Power, Zhengzhou 450046, China; holmelai@163.com (E.Z.);

**Keywords:** wide-row precision sowing, drip irrigation frequency, winter wheat, photosynthetic product accumulation, source–sink coordination, water use efficiency, grain yield, grain quality

## Abstract

To address the issues of low water and fertilizer use efficiency and limited yield potential in traditional winter wheat cultivation in Henan Province, and to determine the optimal drip irrigation frequency for wide-row precision sowing under a constant total irrigation amount, this study was conducted based on a field experiment in Zhengzhou, Henan, during the 2024–2025 season. Four treatments were set up: border irrigation with wide-row precision sowing (QK40), and single-drip irrigation events of 25 mm (DK25, high frequency), 40 mm (DK40, medium frequency), and 55 mm (DK55, low frequency). The effects of drip irrigation frequency on photosynthetic accumulation after anthesis (AUC), source–sink coordination index (SSCI), and the synergy among water, grain, and quality in wide-row precision-sown winter wheat were quantitatively analyzed. The results showed that DK25 significantly delayed leaf senescence and extended the green leaf functional period by 9 days by stabilizing moisture in the 0–40 cm root zone. Post-anthesis photosynthetic accumulation increased by 23.39% and was highly significantly positively correlated with yield. The leaf area index at the heading stage increased by 23.54%, and the source–sink coordination index (SSCI) improved by 45.1%. Over the whole growth period, water consumption was reduced by 10.38%, water use efficiency increased by 23.5%, and yield increased by 8.9%, while grain quality remained stable. Entropy Weight-TOPSIS evaluation showed that DK25 performed the best. This study can provide a cultivation pattern and technical parameters for water-saving, high-yield, and high-quality wide-row precision-sown winter wheat in the Huang-Huai-Hai Plain.

## 1. Introduction

The China’s Henan Province is a major winter wheat production area. In the Huang-Huai-Hai Plain, the over-extraction of groundwater coexists with rigid constraints on irrigation water, posing great challenges to agricultural development. Under the traditional “border irrigation + conventional drill sowing” system, low water and fertilizer use efficiency and limited yield potential have become key obstacles to sustainable wheat production in the region [1]. With the adoption of the national strategy for water-saving agriculture—”determining production based on water availability”—the key question for both research and practical application is how to achieve the synergy of water conservation, high yield, and superior grain quality under limited irrigation conditions [2,3]. Long-term multi-site experiments show that in the northern irrigated wheat zone, water use efficiency (WUE) during the entire growing season is low under conventional border irrigation. Excessive irrigation fails to significantly boost yields; rather, it exacerbates deep percolation and accelerates the decline of the groundwater table [4,5].

Drip irrigation, with its “timed, quantified, and localized” controlled water distribution, has been recognized as a key technological means for water conservation and increased yield in food crops within arid and semi-arid regions. It dramatically lowers surface evaporation and deep percolation losses and improves the root-zone moisture environment [6,7]. Under the condition of constant total irrigation amount, more frequent irrigation with smaller single-event quotas is conducive to maintaining stable soil moisture, improving root water absorption and leaf photosynthetic capacity. This, in turn, enhances WUE and irrigation water use efficiency (IWUE) [8,9]. As an example, in the winter wheat–summer maize rotation zone of northern China, moderate high-frequency drip irrigation can sustain or even elevate yields while reducing water consumption by 10–20% [8,10]. Yet, irrigation timing and single-event volume are crucial factors determining the efficacy of drip irrigation. When the quota is too small or the frequency is improper, water stress may occur during critical growth stages, leading to premature leaf senescence and limited photosynthetic potential. Conversely, excessively large quotas increase ineffective water consumption and may induce lodging [7,9,11]. Thus, optimizing drip irrigation frequency and single-event quota under a constant total irrigation water constraint to develop a “low water, high efficiency” irrigation regime is a core technical issue urgently needing a solution for regional winter wheat cultivation [6,10].

Wide-row precision sowing optimizes the vertical and horizontal structure of the canopy by widening the row spacing, narrowing the seeding band width, and improving seeding uniformity. This enhances the leaf area index (LAI) during the heading stage, improves ventilation and light penetration within the population, and optimizes the pattern of light interception and allocation [12,13]. Recent trials in the Huang-Huai-Hai Plain and other northern irrigated wheat zones have shown that wide-row precision sowing can enhance the efficiency of dry matter accumulation, coordinate the relationship between spike number and grain weight, and enhance WUE and nitrogen use efficiency [14,15]. Under limited irrigation conditions, a reasonable plant-row configuration and an optimal light environment can maintain high population LAI and radiation use efficiency at relatively lower water consumption levels, providing greater regulatory space for the optimization of irrigation regimes [13,16].

Significant interaction effects exist between irrigation methods and planting patterns. Recent field experiments demonstrate that coupling drip irrigation with wide-row precision sowing can optimize canopy structure and photosynthetic function, significantly prolonging the duration of green leaf activity and increasing photosynthetic accumulation. This approach achieves synergistic benefits of water conservation and high yield by reducing total water consumption while enhancing WUE, IWUE, and grain yield [17,18]. Under Huang-Huai-Hai Plain conditions, the combination of “drip irrigation + wide-row precision sowing” can create an efficient photosynthetic status characterized by “high stomatal conductance—low intercellular CO_2_ concentration—high net photosynthetic rate (Pn).” Such a synergy leads to an approximately 20% increase in LAI at the heading stage and a decrease in total seasonal water consumption by about 20%, while yield and protein production increase synchronously [17]. Therefore, the synergistic optimization of precision drip irrigation and wide-row precision sowing is a viable technical pathway to break through the bottlenecks of traditional cultivation and achieve coordinated enhancement in water, grain, and quality for winter wheat [16,17,18].

The temporal variation in water supply and its regulation during critical stages constitute the physiological basis for optimizing drip irrigation frequency. Existing studies indicate that water status during the jointing to grain-filling stages has a strong influence on flag leaf chlorophyll content (SPAD), Pn, the duration of green leaf retention, and the rate of dry matter accumulation, thereby determining the grain-filling process and the formation of thousand-grain weight [19,20]. Although moderate deficit irrigation can improve WUE, excessive deficit or stress during critical stages such as jointing, flowering, and early grain filling is usually at the expense of significant yield loss [4,19,20,21]. Most existing studies largely use instantaneous photosynthetic rates or single-point LAI measurements to describe photosynthetic properties, while quantitative understanding regarding the post-anthesis photosynthetic product accumulation process and its quantitative relationship with source–sink coordination and grain weight formation remains relatively weak [20,22].

Recently, some scholars have incorporated photosynthetic accumulation, source–sink coordination indicators, WUE, yield, and quality into comprehensive evaluations using multi-objective decision-making methods like the entropy weight method and TOPSIS. These approaches are used to evaluate the trade-offs among water, grain, and quality under different irrigation regimes or cultivation patterns to identify optimal management strategies under resource constraints [23]. However, existing studies mostly explore irrigation frequency or planting patterns in isolation. The quantitative regulatory mechanisms of single-event drip irrigation quotas on post-anthesis photosynthetic accumulation, source–sink coordination, and water–grain–quality synergy in wide-row precision-sown winter wheat under the condition of constant total irrigation volume remain unclear [18,21,24]. Particularly in actual production scenarios, there is insufficient field data support to quantitatively identify the optimal combination of water consumption interval and drip irrigation frequency that balances high yield, stable quality, and water conservation [25].

In view of this, this study was conducted within the winter wheat production system of the Huang-Huai-Hai Plain that uniformly adopts wide-row precision sowing. Four treatments were set to systematically monitor key indicators such as soil moisture, water consumption processes, leaf photosynthetic physiology, dry matter accumulation and allocation, source–sink coordination index (SSCI), grain yield, and quality. A comprehensive evaluation framework of “photosynthetic accumulation–source-sink coordination–water-grain-quality” was constructed. The objective of the study is to quantitatively reveal the physiological and ecological mechanisms for the synergistic enhancement of water use, yield, and quality under drip irrigation frequency regulation, identify the optimal drip irrigation frequency for regional promotion, and provide theoretical support and technical parameters for constructing water-saving and high-efficiency cultivation models for winter wheat in the Huang-Huai-Hai Plain.

## 2. Materials and Methods

### 2.1. Experimental Site Description

**Figure 1:**
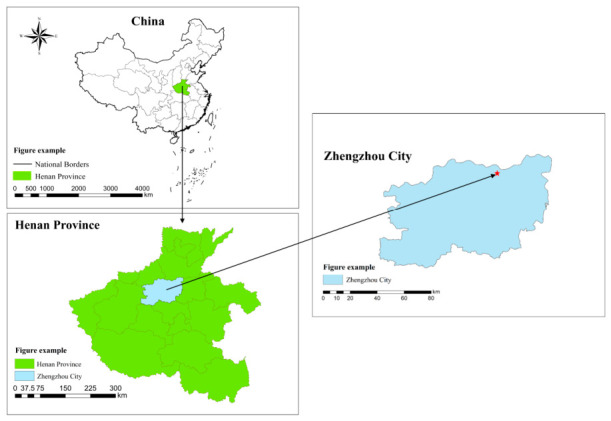
Location of the experimental site.

**Figure 2:**
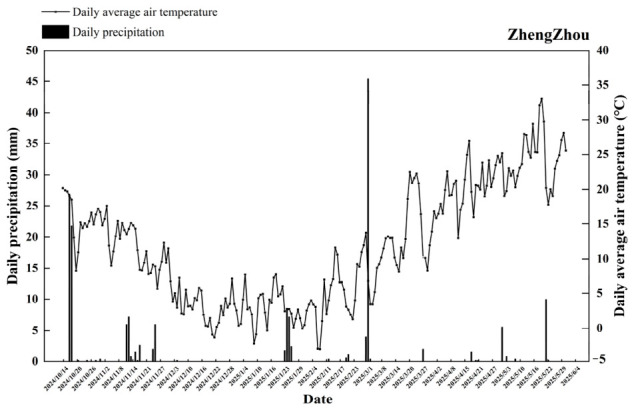
Distribution of temperature and precipitation during the wheat growing seasons.

**Figure 3:**
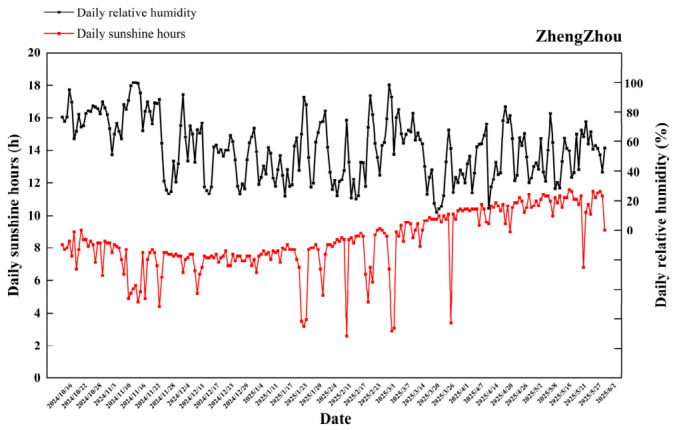
Distribution of relative humidity and sunshine hours during the wheat growing seasons.

The field experiment was conducted from October 2024 to June 2025 in Madu Village, Huiji District, Zhengzhou City, Henan Province (113.7956° E, 34.8665° N). The climate of the experimental site is warm temperate continental monsoon. The mean temperature remained stable at 11.77 °C in the wheat growing season and had a total rainfall of 169.40 mm. The soil type is clay, with a bulk density of 1.56 g/cm^3^ in the 0–100 cm layer and a field water capacity of 28.08% (by weight). Its uniform soil texture renders it for irrigation experiments. Figure 1 displays the geographical position of the place where the experiment was conducted, and Figure 2 reveals the processes of precipitation and air temperature changes. Figure 3 reveals the dynamic processes of relative humidity and sunshine hours. Meteorological data during the experimental period were recorded by an automatic weather station installed in the experimental field, and the data of Leaf Area Index (LAI) were derived from these daily records. The temperature and precipitation conditions throughout the growing season regulated soil moisture fluctuation, which further interacted with different irrigation frequencies to jointly affect leaf senescence, canopy photosynthesis and water consumption characteristics of winter wheat. The meteorological background of this growing season was fully integrated into the subsequent analysis of treatment differences.

### 2.2. Experimental Design

#### 2.2.1. Treatment Setup

The randomized complete block design with one factor and four treatments having three replicates was used to guarantee the reliability of statistical analysis. The specific treatments were determined as follows:

- QK40 (Border Irrigation + wide-row Precision Sowing): Sowing pattern: wide-row precision sowing (band width 8–10 cm, row spacing 25 cm). Irrigation method: Border irrigation with a single application depth of 40 mm
- DK25 (Drip Irrigation + wide-row Precision Sowing): Sowing pattern: wide-row precision sowing (band width 8–10 cm, row spacing 25 cm). Irrigation method: Drip irrigation with a single application depth of 25 mm at high frequency.
- DK40 (Drip Irrigation + wide-row Precision Sowing): Sowing pattern: wide-row precision sowing (band width 8–10 cm, row spacing 25 cm). Irrigation method: Drip irrigation with a single application depth of 40 mm at medium frequency.
- DK55 (Drip Irrigation + wide-row Precision Sowing): Sowing pattern: wide-row precision sowing (band width 8–10 cm, row spacing 25 cm). Irrigation method: Drip irrigation with a single application depth of 55 mm at low frequency.

The total irrigation amount was kept constant across all treatments in this study. Differences in irrigation frequency were established by varying the single-event irrigation quota; that is, a smaller single-event quota corresponded to a higher irrigation frequency.

#### 2.2.2. Experimental Specifications

**Figure 4:**
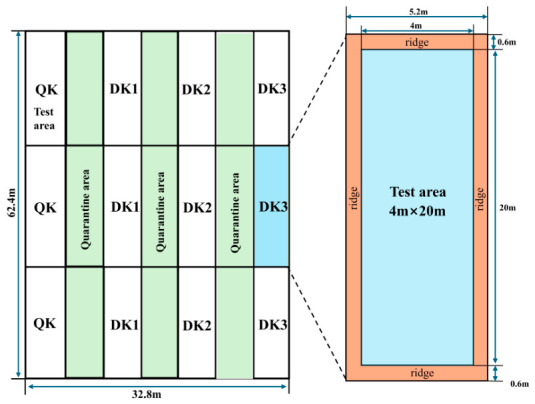
Schematic diagram of the experimental site layout.

**Figure 5:**
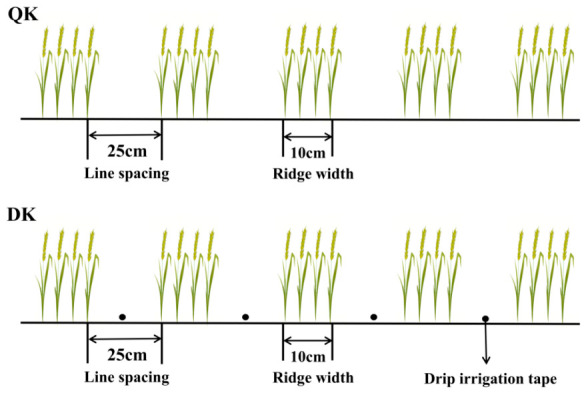
Schematic diagram of seeding width and drip irrigation tape layout.

Each plot measured 20 m in length and 3.5 m in width, with a 3.5 m wide buffer row established between adjacent plots (see Figure 4). Ridges were constructed along the plot boundaries to prevent lateral water movement, and the layout of the drip irrigation tapes. (see Figure 5).

### 2.3. Field Management

#### 2.3.1. Sowing and Fertilization

The experimental variety used was ‘*Bainong 307*’, a high-yielding wheat cultivar widely grown in the Huang-Huai-Hai region. The sowing rate was set at 375 kg/ha. Before sowing, compound fertilizer (N-P_2_O_5_-K_2_O = 15-15-15) was applied as a base dressing at a rate of 120 kg/ha. At the stage of jointing, urea (pure N) was applied as a top-dressing at 150 kg/ha. The fertilization levels were in line with those of local high-yield fields.

#### 2.3.2. Irrigation Management

Irrigation thresholds were based on the requirements to produce high-yield and water-saving wheat in the Huang-Huai-Hai region: irrigation commenced at the stage in which the context of the soil water in the 0–80 cm layer declined to less than 60% of field capacity. Furrow irrigation was done through flood irrigation and drip irrigation was done by using in-line flat tape emitters (emitter spacing 30 cm, flow rate 2.2 L·h^−1^, operating pressure 0.15 MPa). Table 1 explains the particular irrigation schedules. Local drip irrigation rates for winter wheat typically range from 20 to 60 mm. To isolate the effects of irrigation frequency, three application rates were established under a uniform total volume of 200 mm: DK25 (25 mm, high frequency) represents a water-saving model; DK40 (40 mm, medium frequency) serves as a control matching conventional border irrigation; and DK55 (55 mm, low frequency) simulates extensive farmer practices. This 15 mm interval allows for precise quantification of frequency effects on photosynthesis, water use, and yield. All irrigations were triggered when soil moisture in the 0–80 cm profile dropped below 60% of field capacity, primarily during the critical jointing-to-grain-filling period. Adjusting the number of events to maintain the 200 mm total ensured irrigation frequency remained the sole experimental variable.

#### 2.3.3. Pest and Disease Control and Field Management

Weeding was performed manually once during the seedling stage. A 1500-fold dilution of 25% imidacloprid was used to control aphids at the jointing stage. During the grain-filling stage, 0.3% potassium dihydrogen phosphate was sprayed once. All other management practices adopted local standard field protocols.

### 2.4. Measurement Indicators and Methods

#### 2.4.1. Measurement of Growth Indicators

- Leaf Area Index (LAI): Calculated using the formulas: S = A × B × 0.76 ÷ 10,000 and LAI = S × M ÷ 10,000. Each treatment was measured weekly, and the mean value of three replicates was recorded.
- SPAD Value: Measured using a chlorophyll meter (Model IN-YL01). Three points were taken on the flag leaf to get an average value; measurements were done once per week. The measurement frequency and data processing methods were the same as those for LAI.

#### 2.4.2. Photosynthetic Physiological Indicators

- Net Photosynthetic Rate (Pn): Measured by a portable photosynthesis system (Li-6400). Measurements were made at 2 days after flowering. For each treatment, three measurements were taken at each sampling time, and the mean value was calculated.
- Photosynthetic Accumulation after Anthesis (AUC): Since the 35 days post-anthesis constitute the critical grain-filling period for winter wheat in the Huang-Huai-Hai region, the photosynthetic accumulation during this period was used to represent the photosynthetic performance of each treatment. This metric represents the photosynthetic performance of each treatment during the critical grain-filling period. It was calculated as the area under the curve of the photosynthetic rate (Pn) versus time for the first 35 days post-anthesis, using the trapezoidal integration method. The unit is μ × m^−2^ × d^−1^.

#### 2.4.3. Water Indicators

- Soil Water Content: Determined using the oven-drying method. To ascertain the changes in soil samples under different growth stages (0–20, 20–40, 40–60, and 60–80 cm; see Table 2), samples were collected at various levels.
- Inter-row Evaporation: Measured using micro-lysimeters, recording the daily evaporation amounts throughout the growth period.

#### 2.4.4. Yield and Quality

- Yield: At maturity, the number of spikes per 1 m^2^ and the 1000-grain weight were measured to calculate the unit area yield.
- Quality: Protein content and wet gluten content were determined using a grain analyzer.
- Limitation of Quality Assessment: This study evaluated only protein and wet gluten content. Comprehensive processing quality parameters (e.g., test weight, falling number, gluten index, dough rheology) were not measured due to equipment constraints. Thus, while basic nutritional quality stability is confirmed, further research is needed to fully characterize end-use processing potential.

#### 2.4.5. Source–Sink Coordination Index (SSCI)

Source–sink coordination index (SSCI) was optimized and constructed using methods developed in previous studies [26]:

(1) $$\mathrm{SSCI}\;=\;100\;\times \;\frac{{\mathrm{LAI}}_{\mathrm{heading}}\;\times \;\mathrm{GLD}}{\mathrm{SN}\;\times \;\mathrm{TGW}}$$

In the equation, ${\mathrm{LAI}}_{\mathrm{heading}}$ represents the leaf area index at heading; GLD denotes the Green Leaf Duration (days), SN is the number of spikes per unit area (plants/ha), and TGW is the 1000-grain weight (g). The source–sink coordination index (SSCI) is a dimensionless index. A higher value indicates better matching between photosynthetic source strength and grain sink capacity, approaching the optimal population level.

#### 2.4.6. Introduction of Experimental Measuring Instruments

Detailed information on all instruments used in this experiment is presented in Table 3.

### 2.5. Data Processing Methods

#### 2.5.1. Growth Indicators

1. Data processing was performed using Microsoft Excel 2016 for data entry, organization, and basic calculations. The data were tested for normal distribution and homogeneity of variance. After meeting these assumptions, Tukey’s HSD test was used for multiple comparisons at a significance level of *p* < 0.05.
2. Analysis of Variance (ANOVA): SPSS 26.0 and SPSSAU 15.0 was used to analyze the data of single-factor ANOVA. The Tukey HSD test were performed on multiple comparisons (*p* < 0.05).
3. Data Presentation: All results are given as Mean ± Standard Error (Mean ± SE). Graphs were generated using Origin 2021 (v9.8).

#### 2.5.2. Formulas for Core Indicators

1. Soil Water Storage (W):

(2) W=a × γ × h

where a is the soil gravimetric water content; γ is the soil bulk density; and h is the soil layer depth.

2. Water Consumption during Growth Period (TAC):

(3) TAC=SWC+P+I

where SWC is soil water consumption (change in storage); P is precipitation; and I is irrigation amount.

3. WUE:

(4) $$\mathrm{WUE}=\frac{Y}{\mathrm{TAC}}$$

where Y is grain yield; TAC is converted to units of m^3^/ha.

4. Irrigation Water Use Efficiency (IWUE):

(5) $$\mathrm{IWUE}=\frac{Y}{I}$$

where Y is grain yield; I is irrigation amount.

#### 2.5.3. Entropy Weight-TOPSIS Multi-Objective Comprehensive Evaluation

An overall analysis was carried out based on Entropy Weight-TOPSIS method with grain yield, protein content, and WUE as evaluation indicators:

- Construct an original decision matrix and eliminate dimensional differences through range normalization;
- Calculate the entropy value and weights of each indicator to determine objective weights;
- Calculate the Euclidean distances of each treatment from the positive and negative ideal solutions;
- Calculate the relative closeness (comprehensive proximity). A higher closeness indicates better performance of the treatment.

## 3. Results and Analysis

### 3.1. Quantitative Impact of Drip Irrigation Frequency on Photosynthetic Accumulation

#### 3.1.1. Post-Anthesis Net Photosynthetic Rate (Pn)

**Figure 6:**
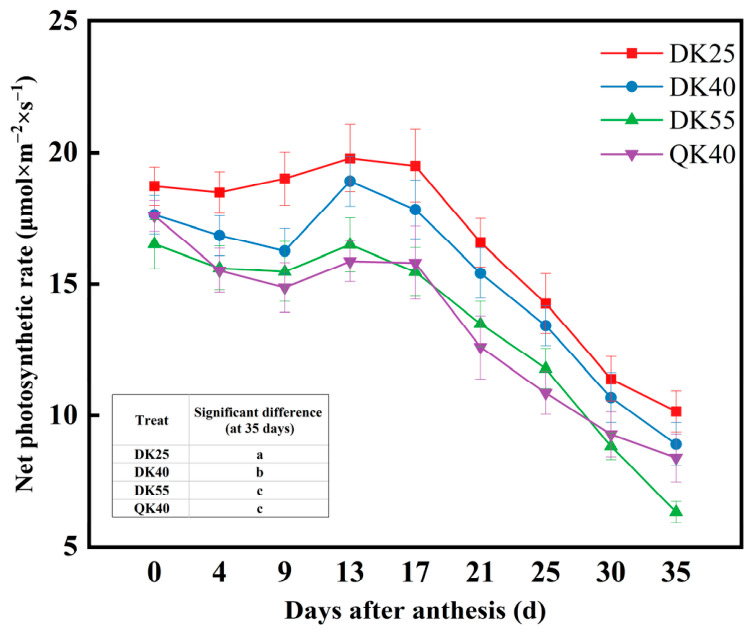
Dynamics of post-anthesis net photosynthetic rate (Pn) in winter wheat under different drip irrigation frequencies (values are mean ± SE; different letters indicate significant difference at *p* < 0.05).

The post-anthesis net photosynthetic rate (Pn) is a direct reflection of photosynthetic product accumulation. In this study, Pn across all treatments showed a unimodal curve (increasing first, then decreasing) during the post-anthesis period, with the peak occurring approximately 13 days after anthesis (see Figure 6). This peak coincides with the vigorous grain-filling stage, indicating that the source–sink relationship is most active at this time to maximize dry matter transport to the developing grains. Combined with the meteorological data in Figure 2, frequent rainfall in mid-May increased relative humidity and reduced daily sunshine hours, which accelerated the natural decline of photosynthetic capacity for all treatments; yet DK25 stabilized root zone water and alleviated this meteorologically induced leaf senescence trend.

However, variations in senescence patterns were observed among treatments. Specifically, QK40 and DK55 exhibited an earlier decline in Pn compared to DK25. This accelerated reduction may be attributed to faster leaf senescence or more efficient remobilization of nutrients from the leaves to the grains, suggesting distinct physiological strategies for carbon assimilation and allocation in these genotypes.

#### 3.1.2. Relationship Between Photosynthetic Accumulation and Yield

**Figure 7:**
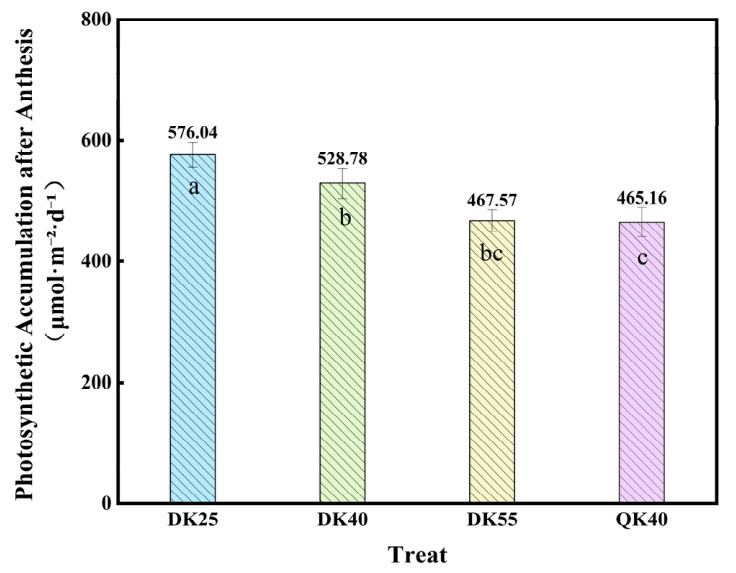
Post-anthesis photosynthetic accumulation (AUC) in winter wheat under different drip irrigation frequencies (values are mean ± SE; different letters indicate significant difference at *p* < 0.05).

**Figure 8:**
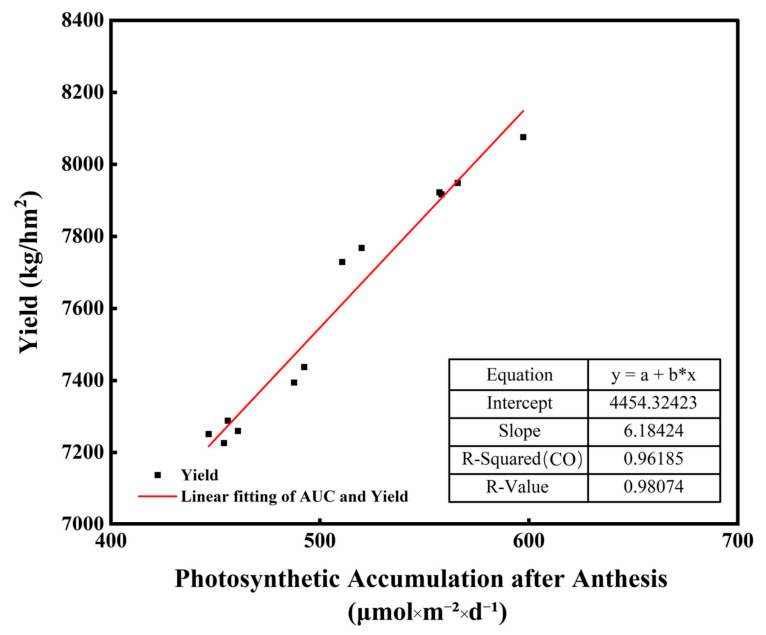
Relationship between photosynthetic accumulation and yield (the data points used in the regression analysis were based on the replicates of each treatment.).

To quantitatively characterize total post-anthesis photosynthetic accumulation, the area under the curve (AUC) was estimated with the trapezoidal integration method. The AUC is an integrated measure of overall contribution of both time and intensity of photosynthesis after anthesis, giving the material basis of yield formation a better indicator than photosynthetic rates at a single time. The results showed that AUC values followed the order: DK25 (576.04 μmol·m^−2^·d^−1^) > DK40 (528.78 μmol·m^−2^·d^−1^) > DK55 (467.57 μmol·m^−2^·d^−1^) > QK40 (465.16 μmol·m^−2^·d^−1^). Specifically, the AUC of DK25 was significantly increased by 23.83% compared to QK40 (Figure 7). Regression analysis indicated that there was a very significant linear positive correlation between AUC and grain yield (R^2^ = 0.96) (Figure 8). This implies that the regulation of yield by drip irrigation frequency is mainly effected by the alteration of the total post-anthesis photosynthetic accumulation. The more photosynthetic accumulation speed, the higher grain production and this forms a consistent quantitative correlation between the two. Thus, post-anthesis photosynthetic accumulation is determined as the key physiological aspect of yield. In addition, the present study sheds light on the quantitative mechanism through which drip irrigation frequency leads to an increase in yield through the control of photosynthetic accumulation. High-frequency drip irrigation stabilizes root-zone water status, delays leaf senescence, amplifies post-anthesis AUC, and is hence the physiological basis behind yield growth.

### 3.2. Quantitative Regulation of Source–Sink Coordination by Drip Irrigation Frequency

#### 3.2.1. Leaf Area Index (LAI)

**Figure 9:**
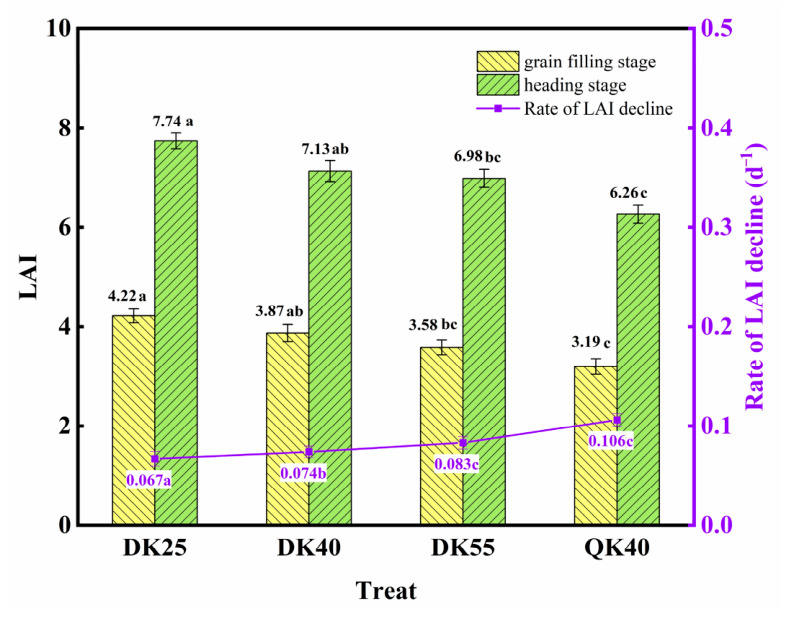
Changes in LAI values during the grain-filling stage and LAI decay rates at the heading stage (values are mean ± SE; different letters indicate significant difference at *p* < 0.05).

The canopy photosynthetic source strength is a fundamental structural measure. The leaf area index (LAI) at heading stage can directly indicate the population light interception rate and photosynthetic area. Experimental data depicted in Figure 9 confirm that, with wide-row precision seeding, drip irrigation frequency has the ability to significantly regulate LAI at heading stage, with an extremely high degree of consistency across years. This shows that the optimizing effect of high-frequency drip irrigation on canopy structure is independent of annual climatic variations or environmental conditions within the trial, implying that this management pattern has constant potential for use.

In the field experiment, LAI at the heading stage followed the order: DK25 (7.74) > DK40 (7.13) > DK55 (6.98) > QK40 (6.26). In particular, the LAI of DK25 was raised by 23.54% in comparison with QK40 and by 10.9% compared to DK55. The increased LAI expanded the canopy photosynthetic area. This, coupled with the ventilation and light-transmission benefits of wide-row precision seeding, helped to avoid canopy closure and consequently greatly enhanced the efficiency of canopy light usage. It can be inferred that the canopy photosynthetic functional period was significantly extended, offering a stable structural base to the accumulation of dry matter after anthesis.

As illustrated in Figure 9, the leaf area index (LAI) during the grain-filling stage exhibited a consistent trend with the heading stage, where the DK25 treatment maintained the highest value (4.22). More critically, the rate of LAI decline differed significantly among treatments. The DK25 treatment demonstrated the slowest decline rate (0.067), which was 9.46% lower than that of DK40 (0.074), 19.28% lower than DK55 (0.083), and 36.79% lower than QK40 (0.106). This indicates that high-frequency drip irrigation effectively mitigates premature senescence during the critical grain-filling period. By maintaining a larger photosynthetic area for a longer duration, the DK25 regime ensures a sustained supply of photo assimilates, thereby providing a robust physiological guarantee for grain weight formation.

The LAI at the heading stage of the DK25 treatment (7.74) was significantly greater compared to other treatments. This substantiates that the effect of high-frequency drip irrigation in optimizing canopy source strength is not dependent on the year or experimental environment, showing very high stability.

According to the mechanism of canopy structure, wide-row precision seeding itself optimizes the ventilation and light conditions of the population. Meanwhile, DK25 high-frequency drip irrigation maintains the root-zone water conditions, favoring the growth and development of wheat leaves. This expands the effective photosynthetic area without canopy closure, accomplishing the formation of a “high-light-efficiency canopy structure.” This is the structural foundation to the photosynthetic physiological advantages found in the DK25 treatment.

#### 3.2.2. Leaf SPAD Values and Green Leaf Functional Period

**Figure 10:**
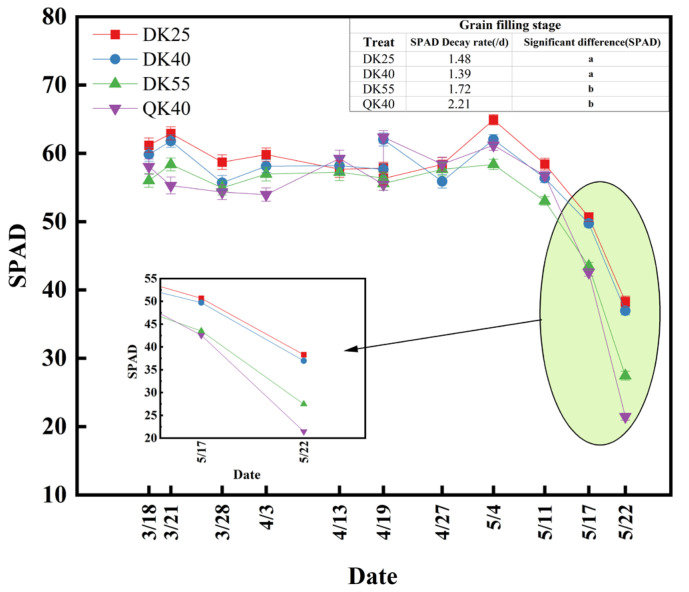
Dynamic changes in leaf SPAD values under different treatments (values are mean ± SE; different letters indicate significant difference at *p* < 0.05).

Leaf SPAD value is a fundamental parameter reflecting chlorophyll content and leaf senescence, with its dynamics directly determining post-anthesis photosynthetic source strength. Figure 10 displays the pattern of seasonal variation in SPAD values across all treatments: rapid increase during the greening stage, stable maintenance during jointing, continuous decline during grain filling, and sharp drop at maturity. The regularity of drip irrigation determines the stability of root-zone water conditions, and thus directly affects the rate of degradation of chlorophyll and the leaf functional period.

Field data show that the grain-filling stage is critical for leaf functional decline. The DK25 treatment had much higher SPAD values than other treatments. On 4 May (early grain filling), the peak SPAD value reached 64.91 for DK25, an increase of 6.08% over QK40 (61.19) and 9.23% over the low-frequency drip treatment DK55 (56.02). By early maturity (22 May), the DK25 SPAD value remained at 38.30, representing a substantial increase of 78.55% compared to QK40 (21.45) and 39.53% compared to DK55 (27.45). This illustrates that high-frequency drip irrigation has great effects on leaf senescence.

Concerning the decay rate, at the stage of grain-filling phase (4 May–22 May), the decline rate of SPAD values for DK25 was only 1.48/d, which was much lower than that of QK40 (2.21/d) and DK55 (1.72/d). Such a low rate of decay guaranteed steady and stable supply of photosynthetic sources throughout the grain-filling period. Despite the slightly lower decay rate of DK40 (1.51/d), it started with a lower value of the first at the start of grain filling than DK25, which led to weaker overall functional maintenance than DK25.

One of the important measures of a prolonged supply of photosynthetic energy is the Green Leaf Duration (GLD). The green leaf functional period was determined through field measurements. It was calculated as the number of days from the onset of the grain-filling stage post-anthesis until the plants completely lost their green leaves. GLD followed the order: DK25 (37 d) > DK40 (34 d) > DK55 (30 d) > QK40 (28 d). Compared to QK40, DK25 extended the green leaf period by 9 days, and by 7 days compared to the low-frequency drip treatment DK55.

The water supply of DK25 is “high-frequency, small-volume”, keeping the soil moisture at 0–40 cm root zone between 60% and 80% of field capacity, without breaking down chlorophylls due to water stress. Conversely, extreme oscillations in soil moisture in the case of furrow irrigation (QK40) and danger of leaching risks associated with low-frequency drip irrigation (DK55) accelerate premature leaf senescence. This reflects the main regulatory impact of irrigation frequency on canopy longevity.

#### 3.2.3. Calculation of Source–Sink Index

Table 4 indicates that the source–sink coordination index (SSCI) between various drip irrigation frequency treatments differs significantly. The increase in the SSCI corresponds to the more reasonable balance between photosynthetic source supply and grain sink capacity that makes the processes of material transportation and grain filling easier. The DK25 treatment exhibited the highest SSCI value of 0.7429, representing a 45.1% increase compared to QK40 (0.5121), and was also superior to DK40 (0.6620) and DK55 (0.5707). This illustrates that DK25 had optimum total strength of leaf area index (LAI) and functional period with respect to its spike density and thousand-grain weight. Conversely, QK40 obtained the lowest SSCI which signifies the scenario in which the “sink” was comparatively too large while “source” supply was insufficient, leading to the worst performance. This source–sink disparity inhibits the transport of photosynthates to grains, eventually lowering the yield. Exploiting the canopy structure through the extension of the green leaf functional period and enhancement of the efficiency of material transport, DK25 achieved a better balance between sources and sinks.

### 3.3. Impact of Drip Irrigation Frequency on Water Use Efficiency

#### 3.3.1. Inter-Row Evaporation

**Figure 11:**
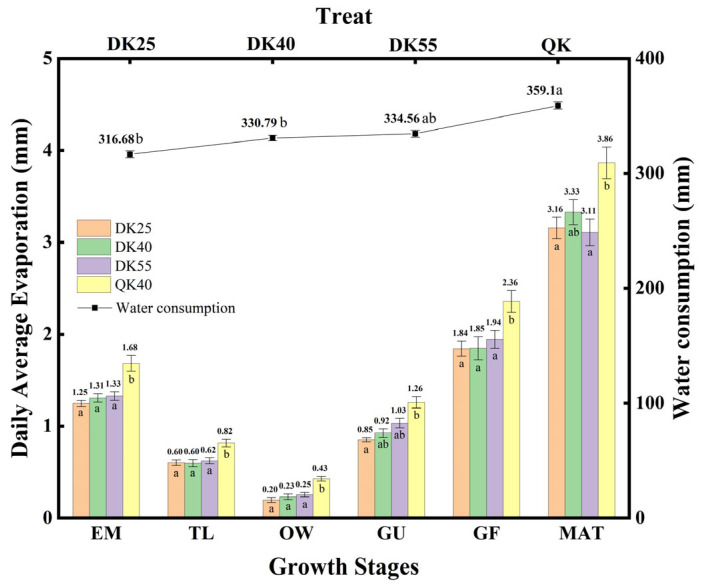
Interannual comparison of inter-row evaporation during the entire growth period of winter wheat under different drip irrigation frequencies (values are mean ± SE; different letters indicate significant difference at *p* < 0.05).

Inter-row evaporation represents a significant portion of ineffective water consumption in farmland. The results from the 2024–2025 field experiment demonstrate that drip irrigation frequency exerts a significant inhibitory effect on this ineffective evaporation. As illustrated in Figure 11, the cumulative inter-row evaporation over the entire growing season followed the order: QK40 > DK55 > DK40 > DK25. This indicates that high-frequency drip irrigation effectively reduces ineffective water loss at the source by minimizing the wetted soil surface area. Notably, the evaporation suppression effect was significantly enhanced as the irrigation volume per event decreased.

Data from key growth stages further corroborate these findings. During the grain-filling stage, the daily evaporation rate in the DK25 treatment was only 1.84 mm/d, representing a 21.8% reduction compared to QK40 (2.36 mm/d) and a 5.2% reduction compared to DK55 (1.94 mm/d). During the green-up and jointing stages, characterized by high soil exposure and intense evaporation, the evaporation rate under furrow irrigation (QK40) was 1.35 to 2.18 times that of DK25. Mechanistically, the “high-frequency, small-volume” strategy of DK25 limits wetting primarily to the crop root zone, thereby minimizing ineffective evaporation from non-root areas and effectuating a transition from “irrigating the land” to “irrigating the crop.” In contrast, both furrow irrigation and low-frequency drip irrigation result in extensive surface wetting and severe ineffective water consumption.

Notably, during the maturity stage (MAT), the inter-row evaporation in the QK40 treatment exhibited an anomalous peak, significantly exceeding that of DK25 and other drip irrigation treatments. This phenomenon is primarily attributed to the disparities in crop population structure and their subsequent impact on the senescence process under different irrigation regimes. The QK40 treatment, employing traditional border irrigation, is characterized by substantial fluctuations in water supply. This instability predisposes the crop to water stress or a decline in root vitality during the late growth stages, leading to the premature yellowing and senescence of flag leaves and the canopy, as well as a rapid attenuation of the leaf area index (LAI). The premature thinning of the canopy exposes a large proportion of the soil surface directly to solar radiation, stripping away the protective shading of the vegetation and thereby inducing intense soil water evaporation during maturity.

Conversely, the DK25 treatment, by maintaining a stable root-zone water potential, effectively delayed leaf senescence (consistent with the aforementioned SPAD results) and sustained a higher green LAI until maturity. The dense canopy formed an effective “biological mulch,” significantly obstructing the pathways of surface heat exchange and water loss. Consequently, the elevated evaporation observed in QK40 during the MAT reflects the inherent limitations of traditional irrigation in terms of poor population quality and weak water retention capacity, further corroborating the superiority of high-frequency drip irrigation in preserving crop ecological functions throughout the entire growth period, particularly in the late stages.

#### 3.3.2. Seasonal Water Consumption and Water Use Efficiency (WUE)

The total seasonal water consumption in the field experiment followed the order: DK25 (316.68 mm) < DK40 (330.79 mm) < DK55 (334.56 mm) < QK40 (359.10 mm). Compared to QK40, the DK25 treatment achieved a 10.38% reduction in water use, while the water-saving rates for DK40 and DK55 were 6.43% and 4.51%, respectively. This demonstrates a clear trend: higher drip irrigation frequency leads to better water-saving effects. By adopting precise, small-volume, and frequent water supply, DK25 concentrates moisture within the highly efficient absorption layer of the root zone. This minimizes deep percolation and field evaporation, resulting in a more rational water consumption structure.

Water use efficiency (WUE) is a core indicator of water–grain synergy. As shown in Table 5, increasing the drip irrigation frequency significantly improved WUE in the field experiments from 2024 to 2025, following the order: DK25 > DK40 > DK55 > QK40. The DK25 treatment achieved a dual enhancement of “lower water consumption and higher yield,” leading to the most significant improvement in WUE. Specifically, the WUE under DK25 reached 2.52 kg/m^3^, representing a 23.5% increase compared to QK40 (2.04 kg/m^3^). Meanwhile, its irrigation water use efficiency (IWUE) was 3.99 kg/m^3^, which was 5.3% higher than that of QK40 (3.79 kg/m^3^).

DK25 achieves the dual advantages of low water consumption and high conversion efficiency through two mechanisms: it reduces ineffective evaporation and deep percolation on one hand, and enhances photosynthesis and source–sink coordination to boost yield on the other, thereby forming a positive feedback loop of “water saving + yield increase.” In contrast, traditional border irrigation is characterized by high water consumption and low water conversion efficiency. Furthermore, due to inappropriate frequencies, low-frequency drip irrigation remains significantly less efficient than high-frequency drip irrigation. These findings confirm that a single irrigation volume of 25 mm represents the optimal drip irrigation frequency for wide-row precision-sown winter wheat. High-frequency, small-volume drip irrigation reduces the soil wetting area, decreases ineffective evaporation and deep percolation, and ultimately realizes the synergy between water conservation and efficient water utilization.

### 3.4. Impact of Drip Irrigation Frequency on Yield and Quality

#### 3.4.1. Yield Components

High wheat yield is based on the synergistic optimization of the three yield components. Field data from 2024 to 2025 show significant differences in spikes per unit area across treatments (Table 4): DK25 (561.33 plants/m^2^) > DK40 (549.64 plants/m^2^) > DK55 (526.88 plants/m^2^) > QK40 (525.37 plants/m^2^). DK25 went up by 6.8% relative to QK40. High-frequency drip irrigation promotes the formation of effective tillers, significantly increases the quantity of effective tillers, and raises the number of effective spikes in the population.

For thousand-grain weight (Table 4), the order was DK25 (45.58 g) > QK40 (45.34 g) > DK40 (45.16 g) > DK55 (44.63 g). DK25 increased by 2.1% than DK55. While increasing the number of spikes, DK25 did not lose its weight, which shows that there was an adequate supply of sources as well as high grain filling degree. There was not the imbalance such as “increasing spikes but reducing grains” or “increasing spikes but reducing weight.” Precise regulation of drip irrigation frequency is the outcome of synergistic optimization of yield components.

#### 3.4.2. Grain Yield and Quality

**Figure 12:**
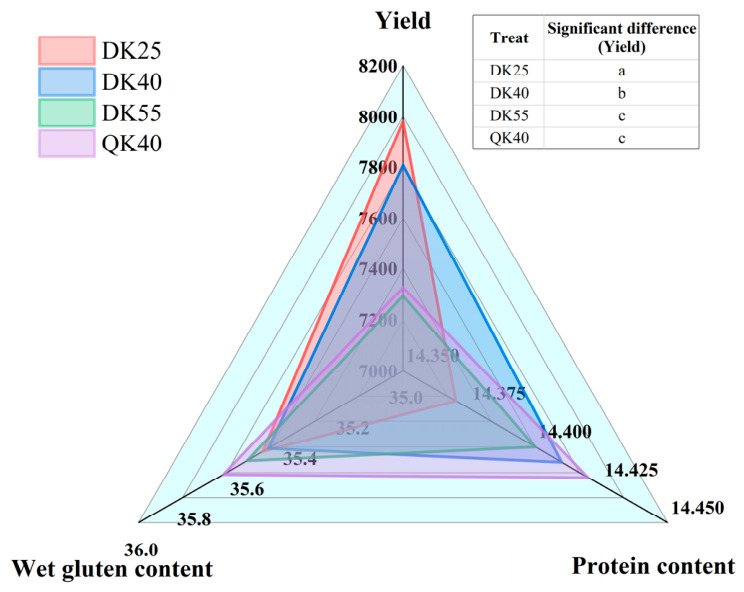
Radar chart of yield and quality under different treatments (values are mean ± SE; different letters indicate significant difference at *p* < 0.05).

The final objective of cultivation management is the high yield of grains. As shown in Figure 12, the ranking of grain yield was DK25 (7980.45 kg/ha) > DK40 (7806.48 kg/ha) > QK40 (7325.62 kg/ha) > DK55 (7293.45 kg/ha). DK25 yielded higher (8.9% and 9.4% better than QK40 and DK55, respectively), indicating a large yield-increasing effect.

Concerning grain quality, no differences in protein content and wet gluten content were found among all treatments. This means that no negative effect on quality of grain was found to affect high-frequency drip irrigation over the range of the irrigation amount used in this experiment. DK25 had a protein content of 14.37% and a wet gluten content of 35.53%, which was basically comparable to QK40 (14.42% and 35.68%). Benefiting from its yield advantage, DK25 had a unit-area total protein production of 1146.79 kg/ha, which is an 8.6% increase over that of QK40. Increase in yield led to an equivalent increase in total protein production and synergy between high yield and stable quality, ensuring simultaneous improvement in yield and stability in quality. This fulfills the production needs of strong-gluten wheat in Huang-Huai-Hai region. High-frequency drip irrigation synergistically optimizes spike number and thousand-grain weight, maximizing yield and overall protein production with no reduction in product quality.

### 3.5. Comprehensive Evaluation of Water–Food–Quality Synergy Under Drip Irrigation Frequency

The Entropy Weight-TOPSIS method was employed to assess the synergistic impacts of water saving, high yield, and high quality at the various drip irrigation frequencies of the 2024–2025 field data (see Table 6). The indicator weights were objectively derived based on the entropy weight method: WUE (0.51) > Yield (0.35) > Protein Content (0.15). This weight distribution shows the production orientation of the Huang-Huai-Hai Plain: “Water saving first, production depending on the availability of water.”

Comprehensive scores showed: DK25 (0.93) > DK40 (0.76) > DK55 (0.46) > QK40 (0.24), with DK25 ranking first. DK25 also performed optimally in two high-weight dimensions of water saving and high yield with constant-quality water, and thus has achieved the full extent of the synergy of water–food–quality improvement. DK25 lacked any deficiencies and was the most balanced among the three objectives of water saving, high yield, and stable quality. QK40 achieved the poorest total because of low WUE and comparatively low yield, quantitatively confirming that DK25 is the optimal cultivation mode for wide-row precision-sown winter wheat.

## 4. Discussion

This study systematically reveals the regulatory mechanisms of drip irrigation frequency on photosynthetic production, source–sink coordination, and the water–grain–quality synergy under the wide-precision sowing mode. The results indicate that high-frequency drip irrigation (DK25) effectively optimizes crop physiological processes by stabilizing the root-zone moisture environment, ultimately achieving synergistic benefits in water saving, high yield, and stable quality. The following discussion delves into the core findings based on the logical sequence of the study.

### 4.1. Regulatory Mechanism of Drip Irrigation Frequency on Photosynthetic Production

This study found that the DK25 treatment exhibited significantly higher net photosynthetic rate (Pn) and photosynthetic accumulation (AUC) post-anthesis, with AUC showing a highly significant positive correlation with grain yield (R^2^ = 0.96). This confirms that regulating the “photosynthetic source” through drip irrigation frequency is the core pathway for yield enhancement under the mode, which aligns with the findings of Li et al. that precise drip irrigation improves crop physiological status and thereby increases yield [27]. The underlying mechanism is that the “small amount, frequent application” water supply strategy of DK25 maintains the soil moisture in the 0–40 cm root layer steadily at 60–80% of field capacity. This stable moisture environment avoids the water stress caused by drastic moisture fluctuations under traditional border irrigation (QK40) and reduces the deep percolation potentially triggered by low-frequency drip irrigation (DK55) [28]. A stable root-zone water supply ensures chlorophyll synthesis in the flag leaf and delays its degradation. This is evidenced by the higher peak SPAD value (64.91) during the grain-filling stage in DK25, with the functional period of green leaves extended by 9 days compared to QK40. The combined effect of a longer photosynthetic functional period and higher photosynthetic intensity significantly increased the total accumulation of post-anthesis photosynthates (AUC increased by 23.39%). This is consistent with the findings of Dong Z. et al. regarding the impact of irrigation frequency on photosynthetic characteristics and dry matter accumulation, thus laying a solid physiological foundation for high yield [29].

In addition, the meteorological conditions (precipitation, sunshine duration and air temperature) of the experimental year created a baseline water environment for wheat growth, and the regulatory effect of high-frequency drip irrigation on photosynthesis was dependent on the coupling relationship between irrigation supply and seasonal meteorological fluctuations. The comprehensive meteorological record of the whole growth period was used to interpret the differential performance of each irrigation treatment. This indicates that low-frequency drip irrigation exacerbates soil moisture fluctuations and accelerates leaf senescence, demonstrating that not all drip irrigation frequencies outperform conventional border irrigation.

### 4.2. Synergistic Optimization of Source–Sink Relationships by Drip Irrigation Frequency

Achieving high yield relies not only on a strong “source” but also on the coordinated development of the “source” and “sink”. This study quantitatively evaluated the coordination status of different treatments using the source–sink coordination index (SSCI). The results showed that DK25 had the highest SSCI value (0.74), an increase of 45.1% compared to QK40. This optimization is achieved through a dual pathway: First, in “source” construction, DK25 promoted wheat canopy development through a stable water supply, resulting in a leaf area index (LAI) of 7.74 at the heading stage, significantly higher than other treatments. The optimized population structure inherent to wide-precision sowing, combined with high-frequency drip irrigation, constructed a “high-light-efficiency canopy,” providing ample space for photosynthesis. This is corroborated by Zhang, M. et al.’s research on the effects of wide sowing on canopy structure [30]. Second, in “sink” formation, an adequate and continuous supply of photosynthates effectively promoted floret differentiation and grain setting, increasing the number of spikes per unit area in DK25 by 6.8% compared to QK40. Crucially, the thousand-grain weight did not decrease despite the increased spike number, indicating that a strong “source” can fully meet the demands of an expanded “sink,” avoiding the source–sink imbalance of “increased spikes but reduced weight,” as described by Ali, S. et al. in their study on source–sink relationships under different irrigation regimes [31]. Therefore, the DK25 mode achieves highly efficient synergy in material production and distribution by simultaneously enhancing source strength and sink capacity to reach an optimal match.

### 4.3. Pathways for Improving Water Use Efficiency via Drip Irrigation Frequency

Against the backdrop of water scarcity in the Huang-Huai-Hai Plain, improving water use efficiency (WUE) is crucial. This study demonstrates that while achieving yield increases, the DK25 treatment saved 10.38% of water and significantly improved WUE by 23.5%. The mechanism for this water-saving and efficiency-enhancing effect is mainly reflected in two aspects: First, reducing ineffective water consumption. High-frequency drip irrigation wets only the crop root zone, substantially reducing inter-plant soil evaporation. Data shows that inter-plant evaporation during the grain-filling stage in DK25 was 21.8% lower than in QK40. Second, optimizing the water consumption structure to improve conversion efficiency. DK25 precisely supplies limited water to critical crop water-demand periods and active root layers, reducing deep percolation. This allows more water to be used for crop transpiration and dry matter accumulation rather than ineffective evaporation and percolation, supporting Yang, D. et al.’s point of view on drip irrigation regulating water use efficiency [32]. This “low-consumption, high-conversion” model breaks the traditional agricultural dilemma where water saving and high yield are mutually exclusive, achieving a fundamental shift from “irrigating the land” to “irrigating the crop,” which aligns with Li, M. et al.’s conclusions on the impact of irrigation strategies on winter wheat WUE [33].

### 4.4. Realization and Comprehensive Evaluation of Water–Grain–Quality Synergistic Effects

The ultimate goal of this study is to achieve synergistic improvements in water, grain, and quality. The results indicate that the DK25 treatment performed best in both grain yield (7980.45 kg/ha, an 8.9% increase) and total protein yield (an 8.6% increase). Meanwhile, grain protein and wet gluten contents remained comparable to the control, successfully achieving a unity of high yield and stable quality. The yield increase stems from the aforementioned enhanced photosynthetic production and optimized source–sink relationships. The stable quality indicates that, within the irrigation volume range of this experiment, high-frequency drip irrigation did not negatively affect the nutritional quality of the grains. Instead, the increase in total biomass drove a synchronous increase in total protein yield, consistent with Kan, Z. et al.’s findings on the responses of winter wheat yield and quality to water–nitrogen coupling [34]. Through the Entropy Weight-TOPSIS comprehensive evaluation, DK25 scored the highest at 0.93, significantly outperforming other treatments. It demonstrated balanced performance across the three dimensions of water saving, high yield, and stable quality, with no obvious shortcomings. The application of this evaluation method references He, P. et al.’s study using the Entropy Weight-TOPSIS method to assess drip irrigation modes [35]. This quantitative evaluation fully proves that the mode paired with high-frequency drip irrigation at 25 mm per application is the optimal technical combination that aligns with the resource endowment and production needs of the Huang-Huai-Hai Plain, as discussed by Qu, X. et al. regarding improving grain quality and nitrogen use efficiency through optimized irrigation management [36].

### 4.5. Research Limitations and Future Perspectives

This study is based on a single-year field trial, which has certain limitations. First, interannual climate variability may have a minor impact on the results, and the generalizability of the conclusions for this specific year requires further validation. Second, the lack of long-term positioning observations means the cumulative effects of different drip irrigation frequencies on soil structure and nutrient evolution remain unclear. Future multi-year trials are needed to verify the stability of these conclusions and explore their long-term comprehensive impacts.

Future research should integrate nitrogen management and planting density optimization to deeply explore the synergistic regulatory effects of multiple factors and their underlying micro-physiological mechanisms. Meanwhile, it is necessary to further validate the universality and stability of this mode by expanding the pilot regions and duration, thereby improving its theoretical framework and supporting large-scale promotion and application.

## 5. Conclusions

DK25 features significantly optimized photosynthetic traits, extending the green leaf functional period by 9 days over QK40 to support high yields. Its source–sink relationship is synergistically improved (LAI + 23.54%, SSCI + 45.1%), effectively balancing supply and demand. The variety reduces water consumption by 10.38% and boosts WUE by 23.5%, achieving “low water use and high conversion.” Field yield increased by 8.9% with stable grain quality, realizing synergistic gains in water, grain, and quality. With a comprehensive score of 0.93 and balanced performance, DK25 is highly adapted to the Huang-Huai-Hai Plain, serving as a reliable variety for water-efficient, high-quality, and high-yielding winter wheat production.

## Figures and Tables

**Table 1:** Irrigation regimes during the growth stages of different treatments.

Irrigation Date	Single Irrigation Amount (mm)			
	QK40	DK25	DK40	DK55
2/28	40	25	40	55
4/11	40	25	40	
4/18		25		55
4/21	40		40	
4/24		25		
4/30	40	25	40	55
5/6		25		
5/12	40	25	40	35
5/24		25		
Irrigation Amount	200	200	200	200

**Table 2:** Correspondence table of growth stages and sampling depths.

Growing Season	Sampling Depth (cm)	Notes
Seedling Stage	0–20	1st Floor
Tillering Stage	0–20	1st Floor
Green-up Stage	0–20,0–40	2nd Floor
Jointing Stage	0–20,0–40,40–60	3rd Floor
Boiling Stage	0–20,0–40,40–60	3rd Floor
Filling Stage	0–20,0–40,40–60,60–80	4th Floor

**Table 3:** Information on major measurement instruments.

Test Item	Instrument Name	Model	Brand/Country of Origin	Accuracy
Plant height, leaf area (length measurement)	Steel tape	—	Deli/Ningbo, China	±0.1 cm
Relative chlorophyll content (SPAD value)	Chlorophyll meter	IN-YL01	Lai Yin Technology/ Changzhou, China	±0.1 SPAD
Photosynthetic parameters (Pn, Gs, etc.)	Portable photosynthesis meter	Li-6400	Li-Cor/Lincoln, NE, USA	Pn: ±0.1 μmol·m^−2^·s^−1^
Grain quality (wet gluten, protein)	Soil moisture content	HM-XM	Hengmei/Nanjing, China	Protein: ±0.1%; Wet gluten: ±0.2%
Soil moisture content (weight measurement)	Electronic analytical balance	FA2004	Ohaus /Parsippany, NE, USA	±0.0001 g
Soil moisture content (drying)	Electric heating constant-temperature oven	DHG-9070A	Jinghong /Nanjing, China	±1 °C
Soil bulk density	Ring knife	100 cm^3^	Nanjing Soil Instruments/Nanjing, China	±0.01 g/cm^3^
Meteorological parameters	Automatic weather station	WX-FGF11H	Wanxiang Technology/ Zhejiang, China	Temperature: ±0.2 °C; rainfall: ±0.1 mm

**Table 4:** Calculation of SSCI values in the field experiment.

Treat	LAI	Green Leaf Duration	Spike Number per Unit Area (Plants/m^2^)	Thousand-Grain Weight (g)	SSCI
DK25	5.14 ± 0.10 a	37 ± 0.37 a	561.33 ± 5.2 a	45.58 ± 0.3 a	0.74 ± 0.02 a
DK40	4.83 ± 0.08 b	34 ± 0.52 b	549.64 ± 4.8 b	45.16 ± 0.2 a	0.66 ± 0.03 b
DK55	4.47 ± 0.07 c	30 ± 0.29 c	526.88 ± 6.1 c	44.63 ± 0.4 b	0.57 ± 0.04 c
QK40	4.35 ± 0.11 a	28 ± 0.64 a	525.37 ± 4.3 a	45.34 ± 0.2 a	0.51 ± 0.03 a

Note: Values are mean ± SE; values followed by different letters are significantly different at *p* < 0.05.

**Table 5:** WUE values under different treatments.

Treatment	Water Consumption/mm	WUE/(kg/m^3^) Water Use Efficiency
QK40	359.10 ± 3.16 c	2.04 ± 0.09 d
DK25	316.68 ± 2.98 a	2.52 ± 0.09 a
DK40	330.79 ± 2.45 b	2.36 ± 0.06 b
DK55	334.56 ± 3.06 b	2.18 ± 0.07 c

Note: Values are mean ± SE; values followed by different letters are significantly different at *p* < 0.05.

**Table 6:** Comprehensive evaluation scores of the 2024–2025 field experiment.

Treatment	Yield Normalization	Protein Normalization	WUE Normalization	Comprehensive Score
QK40	0.00	1.00	0.00	0.24
DK25	1.00	0.00	1.00	0.93
DK40	0.70	0.80	0.67	0.76
DK55	−0.05 ^1^	0.60	0.38	0.46

Note: ^1^ DK55 exhibited a marginally lower yield compared to QK40, leading to a negative value post-normalization. This is an expected outcome in range standardization, signifying performance below the baseline.

## Data Availability

This study utilizes a reliable data set generated from our field experiments. We encourage the academic community to freely reference this resource. The original contributions presented in this study are included in the article. Further inquiries can be directed to the corresponding author.

## Abbreviations

The following abbreviations are used in this manuscript:

SSCI	Source–Sink Coordination Index
AUC	Photosynthetic Accumulation after Anthesis
WUE	Water Use Efficiency
IWUE	Irrigation Water use Efficiency
LAI	Leaf Area Index
SN	Spike Number
TGW	1000-Grain Weight
